# Pharmacological Inhibition of Glycogen Synthase Kinase 3 Regulates T Cell Development *In Vitro*

**DOI:** 10.1371/journal.pone.0058501

**Published:** 2013-03-20

**Authors:** Jan-Hendrik Schroeder, Lewis S. Bell, Michelle L. Janas, Martin Turner

**Affiliations:** Laboratory of Lymphocyte Signalling and Development, The Babraham Institute, Babraham Research Campus, Cambridge, United Kingdom; BSRC 'Alexander FLEMING, Greece

## Abstract

The development of functional T cells requires receptor-mediated transition through multiple checkpoints in the thymus. Double negative 3 (DN3) thymocytes are selected for the presence of a rearranged TCR beta chain in a process termed β-selection which requires signalling via the pre-TCR, Notch1 and CXCL12. Signal integration by these receptors converges on core pathways including the Phosphatidylinositol–3-kinase (PI3K) pathway. Glycogen Synthase Kinase 3 (GSK3) is generally thought to be negatively regulated by the PI3K pathway but its role in β-selection has not been characterised. Here we show that developmental progression of DN3 thymocytes is promoted following inhibition of GSK3 by the synthetic compound CHIR99021. CHIR99021 allows differentiation in the absence of pre-TCR-, Notch1- or CXCL12-mediated signalling. It antagonizes IL-7-mediated inhibition of DP thymocyte differentiation and increases IL-7-promoted cell recovery. These data indicate a potentially important role for inactivation of GSK3 during β-selection. They might help to establish an *in vitro* stromal cell-free culture system of thymocyte development and offer a new platform for screening regulators of proliferation, differentiation and apoptosis.

## Introduction

T cells expressing the αβ T cell receptor (TCR) are formed in the thymus. Progenitors of these cells follow a developmental course as they move through the thymus, starting at the cortico-medullary junction and migrating to the sub-capsular zone (SCZ) prior to returning by this route to enter the medulla for further maturation [1], [2]. Early stage thymocytes are termed double-negative (DN) as they do not express the cell surface glycoproteins CD4 and CD8. DN thymocytes develop into immature CD8^+^ single positive (iSP) cells prior to expression of both CD4 and CD8 that defines the double positive (DP) stage. DP thymocytes subsequently lose expression of CD4 or CD8 to become either CD4^+^ single positive (SP) or CD8^+^ SP mature T cells.

Antigen receptor expression and quality is stringently controlled at specific developmental checkpoints in the thymus. As rearrangement of the TCRβ takes place before that of the TCRα, the first of these checkpoints is referred to as β-selection [1]. The outcome of successful selection at this developmental stage is characterised by extensive proliferation and differentiation from DN to DP. Immediately prior to β-selection, thymocytes can be defined by cell surface staining as CD44^−^CD25^high^CD98^low^CD27^low^ DN3 cells (referred to as DN3a) [3], [4]. Cells in which TCRβ has been rearranged successfully to form a complex with precursor TCR α-chain and CD3 increase in size as well as cell surface expression of CD5, CD98 and CD27 and are referred to as DN3b [3], [4]. Failure to rearrange TCRβ and by consequence to undergo β-selection results in apoptosis [5].

Successful β-selection is not a cell-autonomous process but requires thymic stromal cells which are the source of mediators essential for thymocyte differentiation and proliferation. These include Notch1 ligands and CXCL12, both of which are abundant at the subcapsular zone (SCZ), and interleukin-7 (IL-7) produced by cells at the cortico-medullary junction [6], [7]. At the DN stage IL-7/IL-7receptor (IL-7R) signalling is important for survival and proliferation, but also blocks thymocyte development towards mature αβTCR^+^ T cells [8]. The β-selection checkpoint shows an absolute requirement for pre-TCR and Notch1 signalling [9], [10] and proliferation is augmented by CXCL12 activation of a CXCR4-ras-p110γ pathway [4], [11], [12] reviewed in [13]. However, other receptors and signal transduction pathways further contribute to thymocyte development [14]–[16].

Glycogen synthase kinase 3 (GSK3) is a constitutively active serine-threonine kinase that has two isoforms termed GSK3α and GSK3β [17]. Both GSK3 isoforms phosphorylate target molecules which subsequently results in their ubiquitinylation and degradation [17]. GSK3 has regulatory functions in pathways that include glycogen metabolism, cell-cycle progression, cytoskeletal rearrangement and inflammation [17]. It is also associated with the regulation of canonical Wnt, Hedgehog and Notch signalling, all of which are known to play a role in T cell development [18]–[20]. GSK3 activity is also regulated via phosphorylation and subsequent ubiquitinylation resulting in its degradation [17]. GSK3 may be inactivated by PI3K-mediated signalling [17], [20]. Indeed, PI3K activated through the TCR has been suggested to be a mechanism of GSK3 inactivation in mature T cells [18]. CHIR99021 is a drug that inhibits both GSK3 isoforms without showing inhibitory effects on other kinases tested [21], [22]. This specificity of CHIR99021 is a distinct qualitative difference to other small molecule inhibitors of GSK3 [21], [22]. Therefore, CHIR99021 was used in this study to examine the role of GSK3 in thymocyte development at the β-selection checkpoint using *in vitro* assays.

The OP9-Delta-like 1 (OP9-DL1) stromal cell model is currently popular for the study of *in vitro* T cell development as it is robust and simple to use [23]. However, the OP9-DL1 system is not suitable to analyze the minimal requirements of T cell development. A stromal cell-free system that recapitulates the feature of OP9-DL1 would be a better platform for these investigations and drug testing. The aim of this study was to understand better the mechanism of T cell development and to use this knowledge to improve the stromal cell-free model for T cell development. We report here that pharmacological inhibition of GSK3 using CHIR99021 allowed development of thymocytes in the absence of signalling by the pre-TCR, CXCR4 or Notch1. Furthermore, inhibition of GSK3 at intermediate concentrations of CHIR99021 promoted DN3 differentiation, an effect that is antagonized by IL-7 signalling. Moreover, greater concentrations of CHIR99021, alike high concentration IL-7, allowed the development of CD8^+^ SP thymocytes and suppressed the appearance of DP cells.

## Materials and Methods

### Mice

Six to eight week old C57BL/6 mice were used throughout the study. Rag2 mutant mice were on a C57BL/6 background. All animal husbandry and experimentation were in accordance with UK Home Office regulations and approved by the Babraham Institute Ethical Review Process and the Home Office.

### Cell purification

DN cells were first enriched by incubation with biotinylated anti-CD8α and anti-Ter119 antibodies followed by streptavidin-coated MACS beads and magnetic depletion. Subsequently, cells were stained with a cocktail of antibodies prior to FACS. DN3a and DN3b cells were isolated as negative for CD4/CD8/CD44/CD11b/CD19/NK1.1/Gr-1/γδTCR/Ter119 and as either CD25^high^CD98^low^ (DN3a) or CD25^int^ CD98^high^ (DN3b).

### Cell Culture

OP9 and OP9-DL1 cells were maintained as previously described [24]. 5×10^4^ DN3a or 1.25×10^4^ DN3b cells were cultured in supplemented α-MEM in 96-well plates that had been previously seeded with 4,000 OP9 or OP9-DL1 cells. For stromal cell-free cultures, DN3 cells were cultured in wells that had been coated overnight with 10 µg/ml of recombinant mouse Delta-like 4 (DL4; R&D Systems). In the stromal cell-free cultures DN3 cells were cultured in the presence or absence of recombinant murine CXCL12 (10 nM) and recombinant murine IL-7 (both from PeproTech). In some experiments CHIR99021 (Axon MedChem) was added with the DN3 cells from the start of culture. In other experiments 6-bromoindirubin-30-oxime (BIO; Tocris Bioscience) or sb415286 (Cayman Chemical) was added to the cell culture and in these experiments 2.5×10^4^ instead of 5×10^4^ DN3a had been seeded. Cells were cultured for up to 96 hours before analysis. Numbers of gated lymphocytes were determined by reference to the inclusion of a fixed number of microbeads (Spherotech).

### FACS analysis

Stained single cell suspensions were analysed by FACS using an LSRII (Becton Dickinson) and FlowJo software (Treestar Inc).

### Statistics

Data are presented throughout as the mean and SD of three biological replicates unless it is indicated differently. For statistical analyses containing two groups, data were analyzed using paired Student's t test and P values are shown in the figures.

## Results

### CHIR99021 promotes DN3 development and survival in the absence of Notch1 signalling

**Figure 1:**
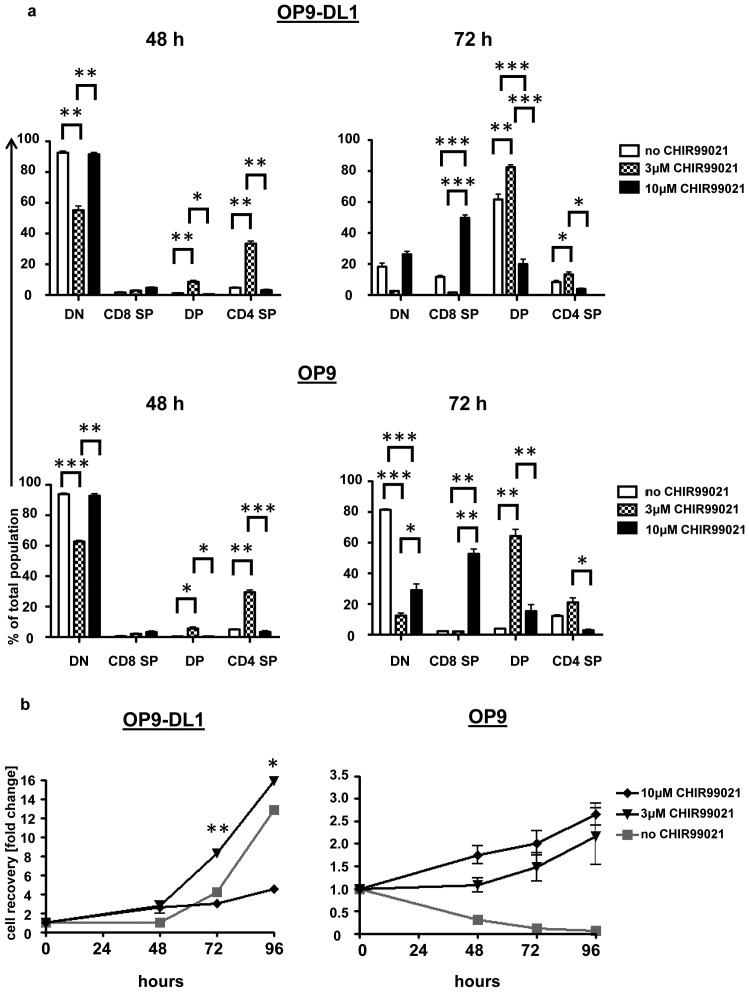
CHIR99021 enhances DN3a development and proliferation in the absence of Notch1 signalling. DN3a cells were cultured on OP9-DL1 or OP9 cells in the presence or absence of CHIR99021 (at 3 or 10 µM). The percentages of CD4^+^CD8^+^, CD4^+^CD8^−^, CD4^−^CD8^+^ and CD4^−^CD8^−^ cells present following 48 and 72 hours of culture (a) and the numbers of cells recovered relative to input at the indicated times points (b) were analyzed. The graph shows the mean and standard deviation of three biological replicates conducted on three different days. The p values were determined using the paired Student's t test (*  =  p<0.05, ** =  p<0.01 and *** =  p<0.001).

We have previously reported that inhibition of GSK3 with CHIR99021 promoted proliferation and differentiation of DN3a thymocytes cultured on plate-bound DL4 in the presence of CXCL12 [11]. To further investigate the effect of CHIR99021, we have used an established model where DN3 cells are cultured on OP9-DL1 stromal cells that are deficient in macrophage-colony stimulating factor (M-CSF) and transduced by a retrovirus expressing DL1 [25]. After 48 hours DN3a-derived thymocyte cultures had hardly expanded but contained some weakly staining DP cells when cultured with 3 µM CHIR99021 (**Figure S1**). Culture with 10 µM CHIR99021 gave rise to fewer DP cells than the lesser concentration, but some CD8^+^cells were found in the cultures. By 72 hours a large proportion of thymocytes present in the drug-free OP9-DL1 cultures were DP. This proportion and the total thymocyte number was enhanced when CHIR99021 was present at 3 µM; at 10 µM, however, there were fewer DP and a significantly greater proportion of CD8^+^ cells which were negative for surface TCR-β expression (**Figure 1** **; Figure S1 and data not shown**). Up to the 72 hour time point 10 µM CHIR99021 did not affect cell recovery significantly in comparison to the control, but at the 96 hour time point cell recovery was reduced (**Figure 1b**).

We compared the effect of GSK3 inhibition on the ability of DN3a cells to proliferate and differentiate in the absence of sustained Notch1 signalling by co-culture with the parental OP9 cell line that does not express DL1 (**Figure 1**). As described previously [9], [26], DN3 cells cultured on OP9 largely fail to differentiate and gradually die upon extended culture. By contrast, when DN3a were co-cultured with OP9 in the presence of 3 µM CHIR99021, differentiation took place. The 10 µM concentration of the drug had little effect on differentiation by the 48 hour time point but by 72 hours a significant number of CD8^+^ and DP cells were present (**Figure 1a** **; Figure S1**). Furthermore, a modest expansion of cell numbers was observed in the presence of CHIR99021 at both concentrations tested. Thymocyte development was not altered by the presence of CHIR99021 at 0.1, 0.3 or 1μM concentrations, while a 30μM concentration of the drug appeared to be toxic to the cells.

**Figure 2:**
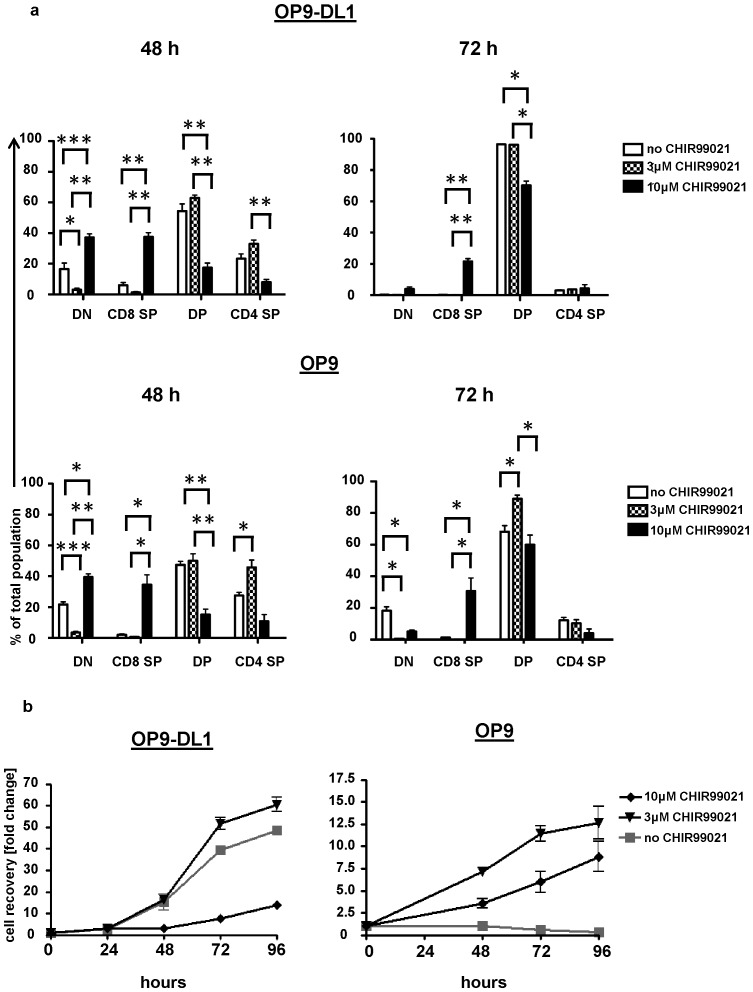
CHIR99021 enhances DN3b development and proliferation in the absence of Notch1 signalling. DN3b thymocytes were cultured on OP9-DL1 or OP9 cells in the presence or absence of CHIR99021 (3 or 10 µM). The percentages of CD4^+^CD8^+^, CD4^+^CD8^−^, CD4^−^CD8^+^ and CD4^−^CD8^−^ cells (a) and the total number of cells harvested relative to input (b) were analyzed. The graph shows the mean and standard deviation of three biological replicates conducted on three different days. The p values were determined using the paired Student's t test (*  =  p<0.05, ** =  p<0.01 and *** =  p<0.001).

The effect of CHIR99021 was also tested on the performance of DN3b thymocytes co-cultured with OP9DL1 or OP9. DN3b cells showed a greater ability to proliferate than DN3a as they expanded 15-fold after 48 hours and approximately 50-fold after 96hours of culture with OP9DL1 (**Figure 2b**). DN3b thymocyte proliferation was Notch1-dependent and there was no expansion when these cells were co-cultured with OP9. However, the DN3b cells did give rise to a predominantly DP cell population when cultured with OP9 suggesting that, under these conditions, sustained Notch1 signalling was dispensable for differentiation. Culture of DN3b cells in the presence of 3 µM CHIR99021 resulted in fewer DN cells in the OP9-DL1 co-cultures after 48 hours, but the extent of differentiation was indistinguishable from the drug-free control by 72 hours (**Figure 2** **; Figure S2**). At 10 µM, CHIR99021 reduced the numbers of cells recovered (compared to 3 µM) and yielded a significantly enhanced proportion of a CD8^+^ population and reduced proportion of DP (**Figure 2** **; Figure S2**). CHIR99021 promoted Notch1 independent DN3b proliferation and, if present at a 3 µM concentration, gave rise to a 10-fold cell expansion in the OP9 co-culture. Taken together, these data indicate that inhibition of GSK3 confers a degree of Notch-1 independence upon DN3 thymocytes.

**Figure 3:**
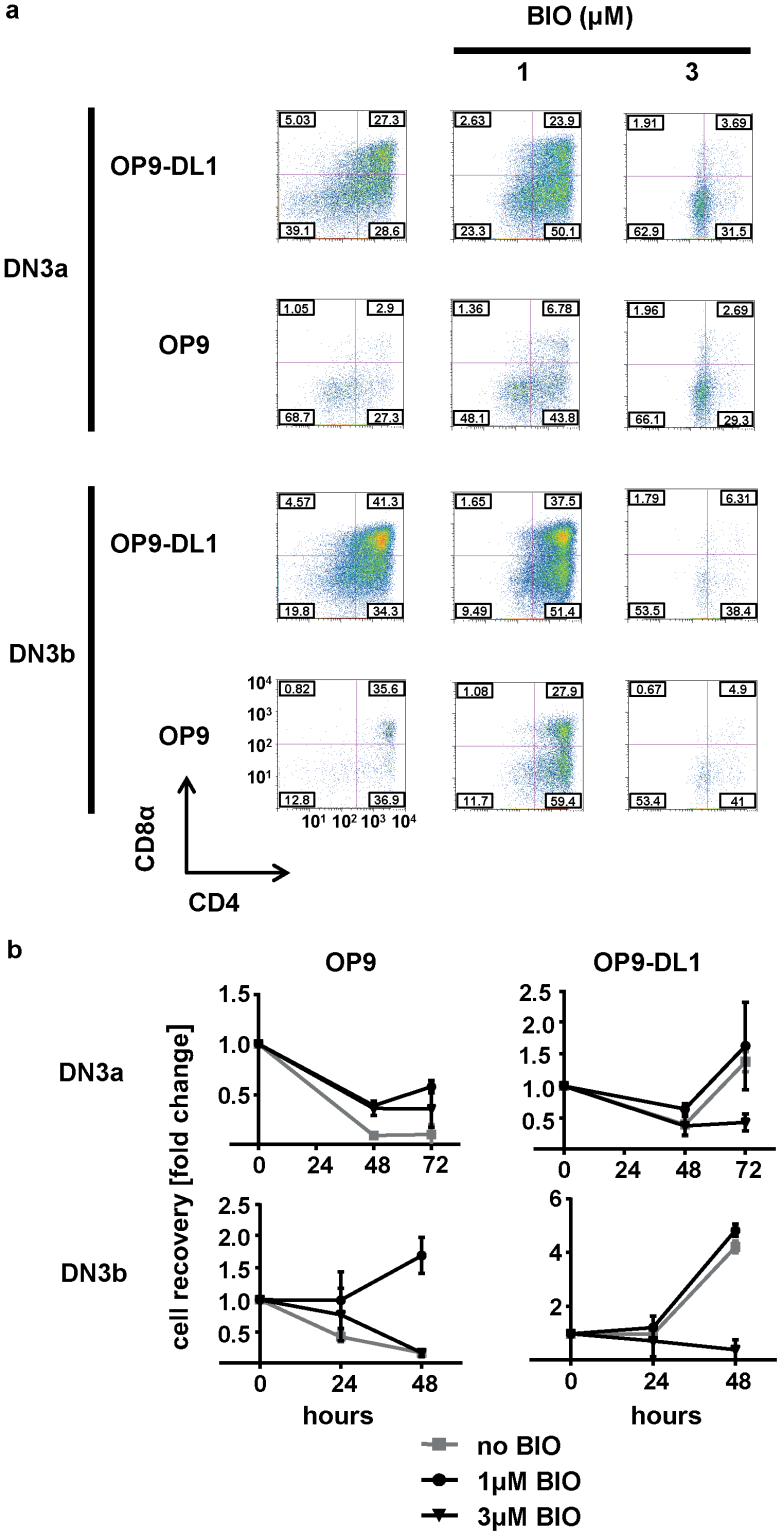
BIO enhances DN3 development and proliferation in the absence of Notch1 signalling. DN3a or DN3b cells were cultured on OP9-DL1 or OP9 cells in the presence or absence of BIO (at 1 or 3 µM). The percentages of CD4^+^CD8^+^, CD4^+^CD8^−^, CD4^−^CD8^+^ and CD4^−^CD8^−^ cells present following 72 hours (DN3a) or 48 hours (DN3b) of culture (a) and the numbers of cells recovered relative to input at the indicated times points (b) were analyzed. The graph shows the mean and standard deviation of two biological replicates conducted on two different days.

We attempted to verify the effects induced by CHIR99021 by using the GSK3 inhibiting drugs BIO and sb415286. In these experiments DN3a or DN3b in co-culture with OP9-DL1 or OP9 were treated with either of these drugs from the start of the culture. The presence of sb415286 in the culture caused an adverse effect on thymocyte development with greatly reduced development of DP and reduced cellularity (data not shown). However, the effects on thymocyte development induced by BIO where similar to those observed upon stimulation with CHIR99021. A 1 µM concentration of BIO appeared to partially mimic the effect induced by 3 µM CHIR99021, whereas a 3 µM concentration of BIO caused similar effects on thymocyte development as 10 µM CHIR99021. In the OP9-DL1 model a 1 µM concentration of BIO resulted in a reduction of DN and promoted the development of CD4^+^ SP derived from DN3a or DN3b cells (**Figure 3a**). The data generated using CHIR99021 were also corroborated by the observation that the same concentration of BIO promoted the development of DP cells derived from DN3a co-cultured with OP9 cells (**Figure 3a**). Furthermore, DN3a or DN3b cells differentiated to CD4^+^ SP in the OP9 model in the presence of 1 µM BIO (**Figure 3a**). In line with the effect induced by CHIR99021, fewer DP cells were present when BIO was included at the higher 3 µM concentration in both stromal cell models (**Figure 3a**). The 1 µM concentration of BIO promoted cellularity of DN3a or DN3b co-cultured with OP9 cells, but was insufficient to enhance cell recovery in the OP9-DL1 model (**Figure 3b**). Furthermore, cellularity of DN3a- and DN3b-derived thymocytes was reduced in the presence of 3 µM BIO in the OP9-DL1 model (**Figure 3b**). 3 µM BIO promoted cell recovery of DN3a-derived thymocytes in the OP9 system, but showed no effect on thymocyte cellularity if DN3b were co-cultured with OP9 stromal cells (**Figure 3b**).

### CHIR99021 promotes DN3 differentiation in the absence of pre-TCR signalling

**Figure 4:**
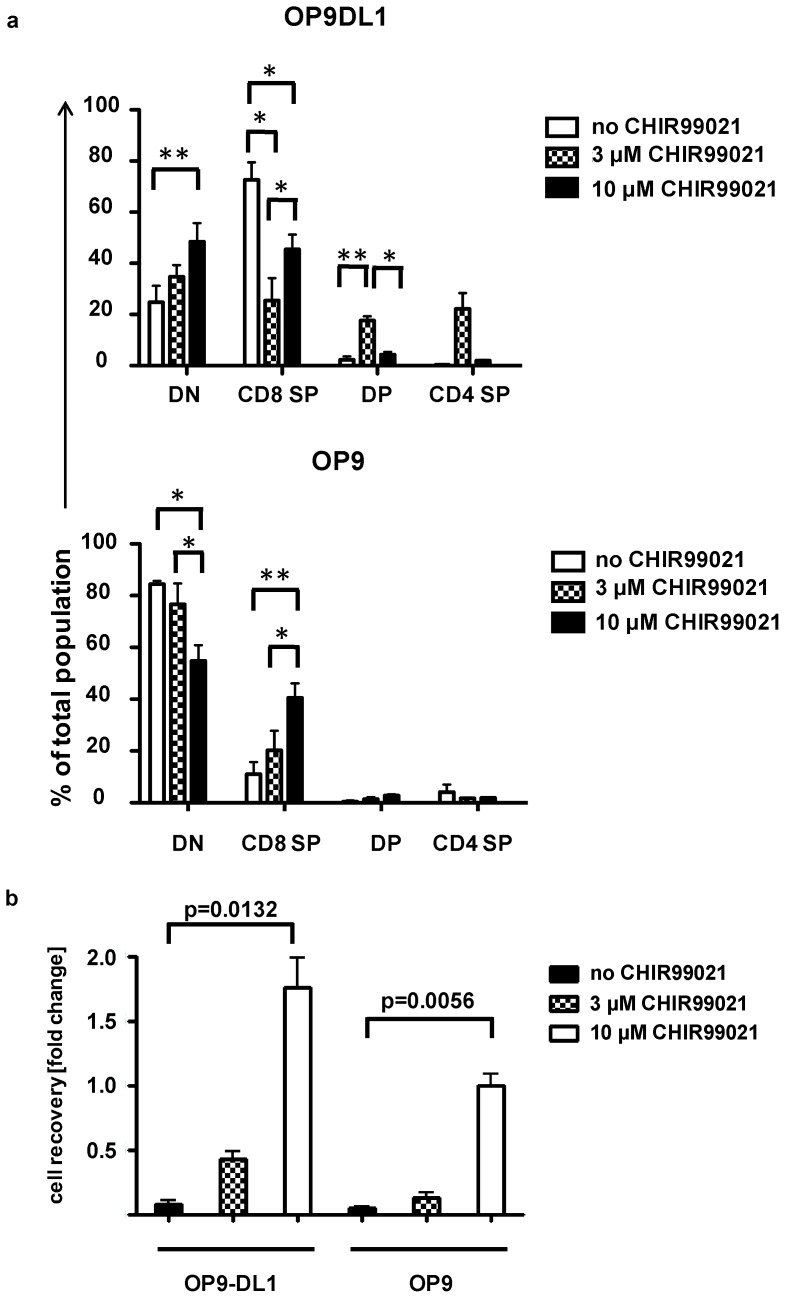
CHIR99021 enhances DN3 development in the absence of preTCR signalling. Rag-2^−/−^ DN3 cells were cultured on OP9-DL1 or OP9 cells in the absence or presence of CHIR99021 (3 or 10 µM). After 48 and 72 hours cells were harvested and analyzed for the percentage of CD4^+^CD8^+^, CD4^+^CD8^−^, CD4^−^CD8^+^ and CD4^−^CD8^−^ cells (a) and at 72 hours the total number of cells harvested relative to input were determined (b). The graph shows the mean and standard deviation of three biological replicates conducted on three different days. The p values were determined using the paired Student's t test (*  =  p<0.05 and ** =  p<0.01).

To investigate whether CHIR99021 could promote differentiation and/or proliferation in the absence of pre-TCR signalling we co-cultured Rag-2-deficient DN3 cells, which lack a pre-TCR and phenotypically resemble DN3a, with OP9-DL1 or OP9 stromal cells in the presence or absence of CHIR99021. Upon co-culture with OP9 Rag-2-deficient DN3 cells did not proliferate and fewer differentiated beyond the DN stage (**Figure 4** **; Figure S3**). By contrast, upon co-culture with OP9-DL1, CD8^+^, but not CD4^+^ or DP, cells were found. In both the OP9-DL1 and OP9 cultures the cell recovery of Rag-2-deficient DN3 cells was much lower than the input cell number, indicating cell death (**Figure 4b**).

Supplementing these cultures with 10 µM CHIR99021 enhanced cell recovery in both stromal cell systems, but cell numbers were always much lower than those observed upon co-culture of Rag-sufficient DN3a cells with OP9-DL1. At the higher concentration of CHIR99021, Rag-2-deficient thymocytes differentiated to CD8^+^ in both OP9 and OP9-DL1 co-cultures (**Figure 4** **; Figure S3**). At the 3 µM concentration of CHIR99021, CD4^+^ and DP thymocytes were observed when Rag-2-deficient DN3a were cultured with OP9-DL1, but the same effect was not observed in the OP9 system (**Figure 4** **;** **Figure S3**). Overall, CHIR99021 shows some ability to rescue DN3 differentiation and survival in the absence of pre-TCR signalling and this is enhanced by sustaining Notch-mediated signalling.

### CHIR99021 enhances DN3 proliferation in the absence of CXCL12

**Figure 5:**
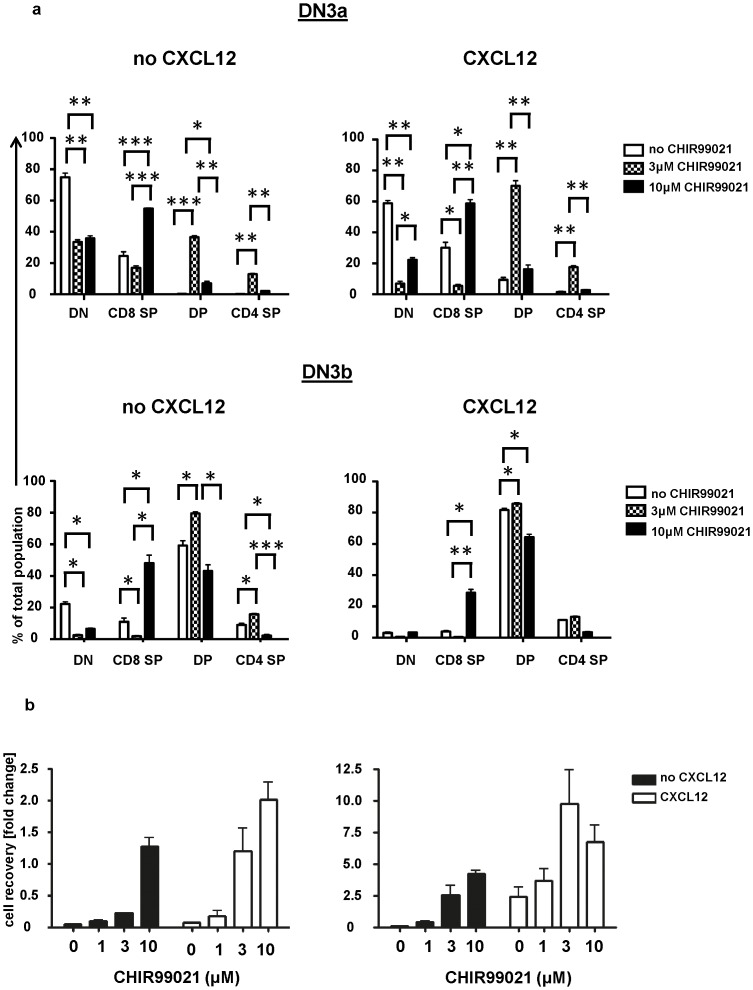
CHIR99021 enhances DN3 development in the absence of CXCL12. DN3 were cultured on plate-bound recombinant DL4 in the presence or absence of CXCL12 (10 nM) and CHIR99021 (1, 3 or 10 µM). After 72 hours cells were harvested and analyzed for the percentages of CD4^+^CD8^+^, CD4^+^CD8^−^, CD4^−^CD8^+^ and CD4^−^CD8^−^ cells (a) and the total number of cells relative to input (b). The graph shows the mean and standard deviation of three biological replicates conducted on three different days. The p values were determined using the paired Student's t test (*  =  p<0.05, ** =  p<0.01 and *** =  p<0.001).

We previously reported that CXCL12 promotes the proliferation of DN3 thymocytes [4], [11]. To examine this further we compared the effect of CXCL12 on DN3a and DN3b thymocytes in stromal cell-free cultures using recombinant DL4 as the source of Notch1 ligand. DN3a cells failed to thrive in the presence or absence of CXCL12 compared to the OP9-DL1 model, but did yield a fraction of DP in a CXCL12 dependent manner (**Figure 5a** **; Figure S4**). Most DN3b cells died in the absence of CXCL12, but, in its presence they proliferated and had expanded 2.5-fold by 72 hours (**Figure 5b**).

We further analyzed the effect of CHIR99021 in this stromal cell-free model. Here we could conclude that effects of the drug were mediated directly on the thymocytes and not via effects on the stromal cells. In the absence of CXCL12, CHIR99021 promoted differentiation of DN3a and DN3b cells and the same effect was seen when DN3a were seeded in the presence of CXCL12 (**Figure 5a** **; Figure S4**). Once again we observed qualitative differences between the effect of the drug at 3 and 10 µM both in the absence and presence of CXCL12. If CHIR99021 was present at 10 µM a higher proportion of CD8^+^ was found (**Figure 5** **; Figure S4**). Furthermore, CHIR99021 promoted the expansion of both DN3a- and DN3b-derived thymocytes and this effect was always greater when CXCL12 was present (**Figure 5b**). In contrast to effects seen in the OP9-DL1 co-cultures, a 10 µM concentration of the drug used in the stromal cell-free system did not inhibit proliferation.

### A high concentration of CHIR99021 like IL-7 antagonizes DN3 cell differentiation

**Figure 6:**
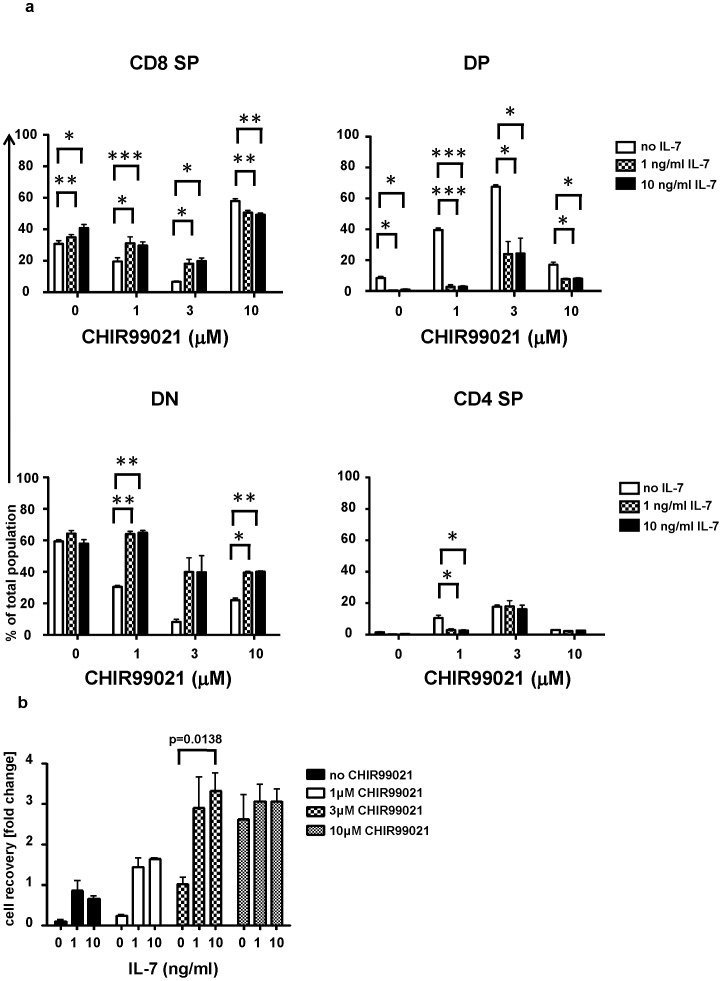
Effects of CHIR99021 and IL-7 on DN3a cell development. DN3a cells were cultured on plate-bound recombinant DL4 in the presence of CXCL12 (10 nM) and the presence or absence of the indicated concentrations of CHIR99021 (1, 3 or 10 µM) and recombinant murine IL-7 (1 or 10 ng/ml). The percentage of CD4^+^CD8^+^, CD4^+^CD8^−^, CD4^−^CD8^+^ and CD4^−^CD8^−^ cells after 72 hours (a) and the total number of cells relative to input (b). The graph shows the mean and standard deviation of three biological replicates conducted on three different days. The p values were determined using the paired Student's t test (*  =  p<0.05, ** =  p<0.01 and *** =  p<0.001).

As IL-7 has been reported to inhibit thymocyte differentiation beyond the DN stage *in vitro* [8], we next examined the effect of adding IL-7 together with DL4 and CXCL12. IL-7 induced a reduction of the proportions of DP cells derived from CHIR99021-stimulated DN3a cells in the cultures (**Figure 6a** **; Figure S5**). The addition of IL-7 to cultures of DN3a cells marginally reduced the loss of cells as well. Furthermore, IL-7 enhanced cell recovery of DN3a-derived thymocytes in combination with CHIR99021 at 1 and 3 µM (**Figure 6b**).

**Figure 7:**
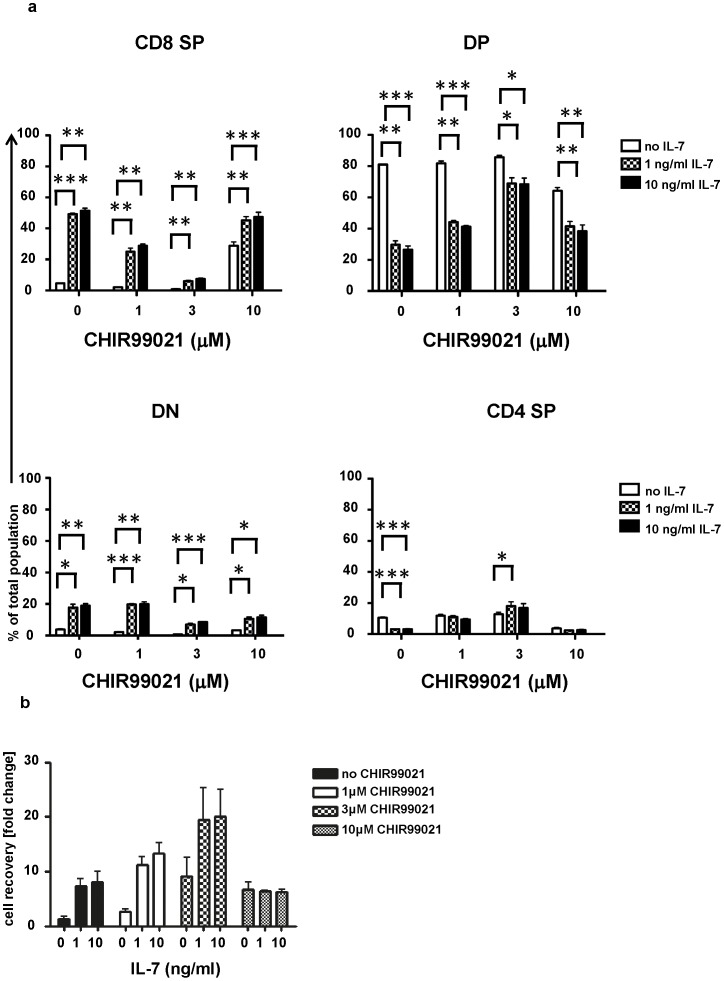
CHIR99021 and IL-7 effects on DN3b development after beta-selection. DN3b cells were cultured as described in the legend to figure 5. The percentage of CD4^+^CD8^+^, CD4^+^CD8^−^, CD4^−^CD8^+^ and CD4^−^CD8^−^ cells following 72 hours culture (a) and the total number of cells harvested relative to input (b) were determined. The graph shows the mean and standard deviation of three biological replicates conducted on three different days. The p values were determined using the paired Student's t test (*  =  p<0.05, ** =  p<0.01 and *** =  p<0.001).

When DN3b cells were cultured with DL4 and CXCL12, IL-7 increased the cellularity of the resulting culture by 7–8 fold (**Figure 7b** **)** and enhanced the presence of CD8^+^ SP and DN cells in these cultures **(** **Figure 7a** **; Figure S6**). IL-7 thus retards the differentiation of DN3b while promoting proliferation. Inclusion of 1 or 3 µM CHIR99021 did not increase the cellularity of the cultures further, but resulted in greater proportion of SP and DN cells (**Figure 7b** **;** **Figure S6**). Thus, these concentrations of CHIR99021 are permissive for differentiation even when IL-7 is present. At the highest concentration of CHIR99021, differentiation was retarded and IL-7 further enhanced the proportion of DN or CD8^+^ SP cells (**Figure 7** **;** **Figure S6**). 10 µM CHIR99021 was less effective at promoting the expansion of DN3b than 3 µM CHIR99021. IL-7 did not affect cell recovery of DN3a- or DN3b-derived thymocytes when CHIR99021 was present at 10 µM.

### CHIR99021 at 10μM enhances CD127 surface expression

**Figure 8:**
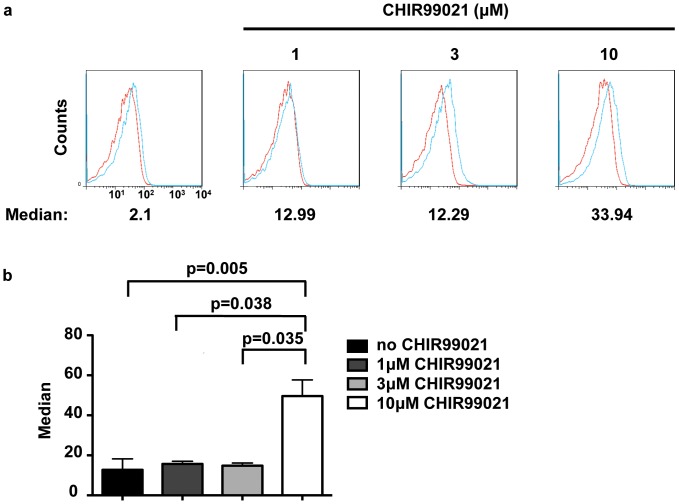
A high concentration of CHIR99021 enhances CD127 surface expression on thymocytes. DN3a were cultured on OP9-DL1 cells in the absence or presence of CHIR99021 (1, 3 or 10 µM). Cells were harvested and stained with either rat IgG2a (shown in red) or rat anti-mouse CD127 (IgG2a; shown in turquoise). Cell surface expression of CD127 on DN3 cells (CD25^+^CD98^+^) at 48 hours was analysed. The median values given for the surface expression (a) and the the graph shows the mean and standard deviation of three biological replicates conducted on three different days. The p values were determined using the paired Student's t test.

The contrasting results obtained following culture of thymocytes with CHIR99021 at 3 and 10 µM led to the hypothesis that, at the higher concentration of the drug, differentiation to the DP stage is inhibited. IL-7 and high concentrations of CHIR99021 both caused the increased appearance of CD8^+^ SP thymocytes in our cultures. We noted that 10 µM but not 1 µM or 3 µM CHIR99021 promoted expression of the IL-7Rα subunit (CD127) on DN3 co-cultured with OP9-DL1 (**Figure 8** **)**. Thus, the higher concentration of CHIR99021 may selectively increase IL-7 signalling and subsequently suppress differentiation while a reduced concentration may favour differentiation.

## Discussion

The data presented in this study are consistent with the hypothesis that the modulation of GSK3 activity is a component of the signalling cascade that promotes the development of DN thymocytes. This study has been performed using the GSK3- inhibiting drugs CHIR99021 and BIO. At present it cannot be entirely excluded that these chemical compounds target additional signalling components leading to the effects presented in this study. However, CHIR99021 has been well studied and is also known to lack effects on other protein kinases. Other commercially available GSK3-inhibiting chemical components have either many side effects or are less well documented. Therefore, CHIR99021 can be considered as the “gold standard” of drug-based inhibition of GSK3. Although our studies have been performed exclusively with this small molecule inhibitor, GSK3 activity may naturally be inhibited following signal transduction by the pre-TCR, Notch1 and/or CXCR4. Each of these pathways contributes to the differentiation and proliferation of thymocytes subsequent to the successful rearrangement and expression of the TCR-β chain. This may be achieved, in part, by the activation of the PI3K system which is a pathway with the potential for inhibition of GSK3 under specific circumstances [27].

This view is in line with the finding that when thymocytes were cultured in the absence of Notch1 or CXCR4 stimulation, pharmacological inhibition of GSK3 by 3 µM CHIR99021 promoted differentiation. Thymocyte differentiation in the absence of the pre-TCR was less affected by inhibition of GSK3, possibly reflecting a requirement for further TCRβ-dependent signalling pathways for efficient development. However, limited differentiation may be able to take place without the pre-TCR as we observed that Rag-2-deficient DN3 cells differentiated into CD8^+^ thymocytes when cultured with OP9-DL1 without added IL-7. It was reported previously that FACS sort-purified Rag-2^−/−^ DN3 cells do not differentiate beyond the DN stage in an OP9-DL1 model when IL-7 is present [9]. This apparent difference may be resolved by the observation that IL-7 causes thymocyte development to arrest at the DN stage [8].

Unlike the differentiation promoting effects of a 3 µM concentration of CHIR99021, the effects of CHIR99021 at 10 µM led to less thymocyte differentiation to DP cells. The nature of the CD8^+^ thymocytes appearing in cultures supplemented with 10 µM CHIR99021 remains unclear. These cells do not express TCRβ and could be related to immature single-positives; cells in transit between the DN and DP compartments. However, expression of CD24 by these cells was lower than iSP derived from OP9-DL1 cultures in the absence of CHIR99021 (data not shown). Moreover, unlike iSP, these CD8^+^ cells express CD127 (data not shown) and their presence was enhanced by stimulation with IL-7 in the stromal cell-free model. It is possible that the development of these thymocytes is retarded by the drug, but it appears unlikely that this concentration of the drug is toxic to the cells as there was always higher cell recovery compared to the initial seeding number.

Our results with 10 µM CHIR99021 bear comparison to studies of transgenic mice where IL-7 was highly expressed in the thymus and maintained in DP thymocytes [28]. These mice had fewer overall thymocytes, a higher proportion of CD8^+^ thymocytes and fewer CD4^+^and DP thymocytes. At the lower concentrations of CHIR99021 tested, CD127 surface expression was not enhanced on DN3a-derived thymocytes (Figure 8) and this might be related to the ability of thymocytes to differentiate efficiently into DP at those concentrations of CHIR99021. We speculate that the concentration dependent effect of GSK3 inhibition on the IL-7R may be the basis for the antagonizing effect of IL-7 on CHIR99021-promoted thymocyte differentiation. Although the IL-7R has not been reported to be phosphorylated by GSK3, there are three S/TxxxS/T motifs, potential phosphorylation sites for GSK3, within the cytoplasmic domain of mouse CD127 which is consistent with the hypothesis that CD127 might be a direct target of GSK3 [29]. However, it seems likely that GSK3 acts through modulating the activity of multiple pathways. Targets of GSK3 which may be relevant to the effects that were manifested in culture might be Notch1, the activity of which may be sustained by inhibition of GSK3 [18]. The canonical β-catenin pathway, which is suppressed by GSK3, has also been shown promote β-selection and when constitutively activated can bypass the requirement for preTCR and Notch1 signals [16], [30]. Potentially, this might explain the ability of cells to differentiate and expand on OP9. Other relevant targets suppressed by GSK3 include c-myc and mcl-1, which may affect proliferation and survival [31].

As the cortico-medullary junction is the location where IL-7 is abundantly expressed, the concentration of IL-7 is likely to be much lower in the subcapsular zone (SCZ) where the expression of DL4 and CXCL12 are highest and β-selection and its associated differentiation events are occurring. Early thymocyte progenitors entering the thymus at the cortico-medullary junction receive signals that promote their growth and survival but block differentiation while they are migrating towards the SCZ. Once thymocytes arrive at the SCZ the IL-7-mediated block in differentiation is weak and can be effectively antagonized by Notch-, CXCR4- and pre-TCR-mediated signalling. Inhibition of GSK3 may be part of the β-selection checkpoint. We hope that our studies using pharmacological inhibition and *in vitro* systems will prompt more definitive studies using conditional mutagenesis.

## Supporting Information

Figure S1 **CHIR99021 enhances DN3a development and proliferation in the absence of Notch1 signalling.** DN3a cells were cultured on OP9-DL1 or OP9 cells in the presence or absence of CHIR99021 (at 3 or 10 µM). The percentages of CD4^+^CD8^+^ (DP), CD4^+^CD8^−^ (CD4 SP), CD4^−^CD8^+^ (CD8 SP) and CD4^−^CD8^−^ (DN) cells present following 48 and 72 hours of culture were analyzed.
(TIF)

Figure S2 **CHIR99021 enhances DN3b development and proliferation in the absence of Notch1 signalling.** DN3b thymocytes were cultured on OP9-DL1 or OP9 cells in the presence or absence of CHIR99021 (3 or 10 µM). The percentages of CD4^+^CD8^+^ (DP), CD4^+^CD8^−^ (CD4 SP), CD4^−^CD8^+^ (CD8 SP) and CD4^−^CD8^−^ (DN) cells were analyzed.
(TIF)

Figure S3 **CHIR99021 enhances DN3 development in the absence of preTCR signalling.** Rag-2^−/−^ DN3 cells were cultured on OP9-DL1 or OP9 cells in the absence or presence of CHIR99021 (3 or 10 µM). After 72 hours cells were harvested and analyzed for the percentage of CD4^+^CD8^+^ (DP), CD4^+^CD8^−^ (CD4 SP), CD4^−^CD8^+^ (CD8 SP) and CD4^−^CD8^−^ (DN) cells.
(TIF)

Figure S4 **CHIR99021 enhances DN3 development in the absence of CXCL12.** DN3 were cultured on plate-bound recombinant DL4 in the presence or absence of CXCL12 (10 nM) and CHIR99021 (1, 3 or 10 µM). After 72 hours cells were harvested and analyzed for the percentages of CD4^+^CD8^+^ (DP), CD4^+^CD8^−^ (CD4 SP), CD4^−^CD8^+^ (CD8 SP) and CD4^−^CD8^−^ (DN) cells.
(TIF)

Figure S5 **Effects of CHIR99021 and IL-7 on DN3 cell development.** DN3a cells were cultured on plate-bound recombinant DL4 in the presence of CXCL12 (10 nM) and the presence or absence of the indicated concentrations of CHIR99021 (1, 3 or 10 µM) and recombinant murine IL-7 (1 or 10 ng/ml). The percentage of CD4^+^CD8^+^ (DP), CD4^+^CD8^−^ (CD4 SP), CD4^−^CD8^+^ (CD8 SP) and CD4^−^CD8^−^ (DN) cells was analysed after 72 hours.
(TIF)

Figure S6 **Effects of CHIR99021 and IL-7 on DN3 cell development.** DN3b cells were cultured on plate-bound recombinant DL4 in the presence of CXCL12 (10 nM) and the presence or absence of the indicated concentrations of CHIR99021 (1, 3 or 10 µM) and recombinant murine IL-7 (1 or 10 ng/ml). The percentage of CD4^+^CD8^+^ (DP), CD4^+^CD8^−^ (CD4 SP), CD4^−^CD8^+^ (CD8 SP) and CD4^−^CD8^−^ (DN) cells was analysed after 72 hours.
(TIF)
