# Correction: Pharmacological Inhibition of Glycogen Synthase Kinase 3 Regulates T Cell Development *In Vitro*

**DOI:** 10.1371/annotation/851be907-9f62-420c-92b8-c31681c3bcbe

**Published:** 2013-05-17

**Authors:** Jan-Hendrik Schroeder, Lewis S. Bell, Michelle L. Janas, Martin Turner

There were errors in Figure 5 and Supporting Figures S4, S5, and S6. The correct versions of these Figures can be viewed here:

Figure 5: 

**Figure:**
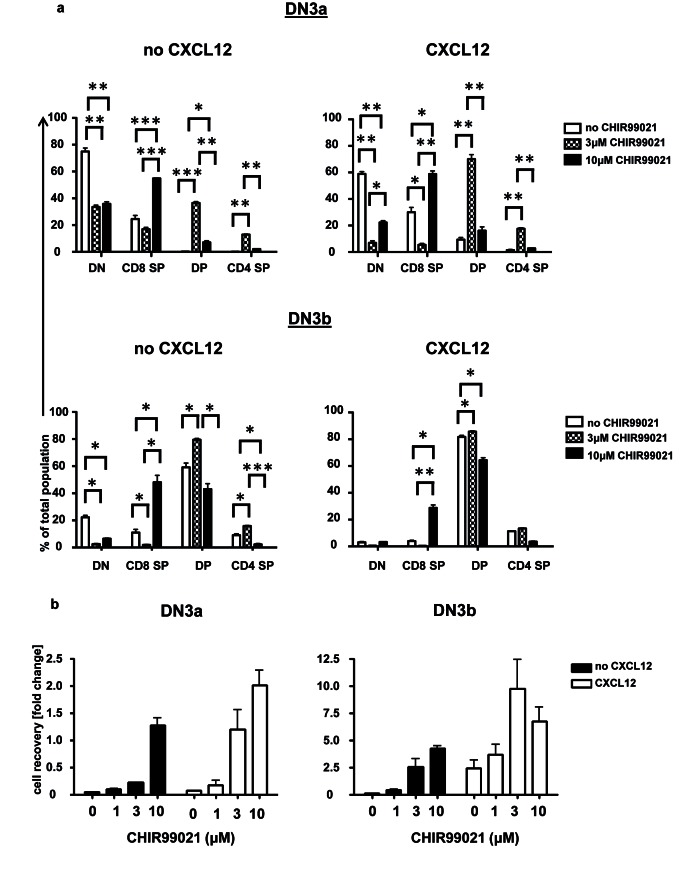


Supporting Figure S4: 

Supporting Figure S5: 

Supporting Figure S6: 
