# Supplementary figures and images for: Pharmacological Inhibition of Glycogen Synthase Kinase 3 Regulates T Cell Development In Vitro
Source: PLoS One. 2013 Mar 20;8(3):e58501. doi: 10.1371/journal.pone.0058501 (PMC3603984; doi:10.1371/journal.pone.0058501)

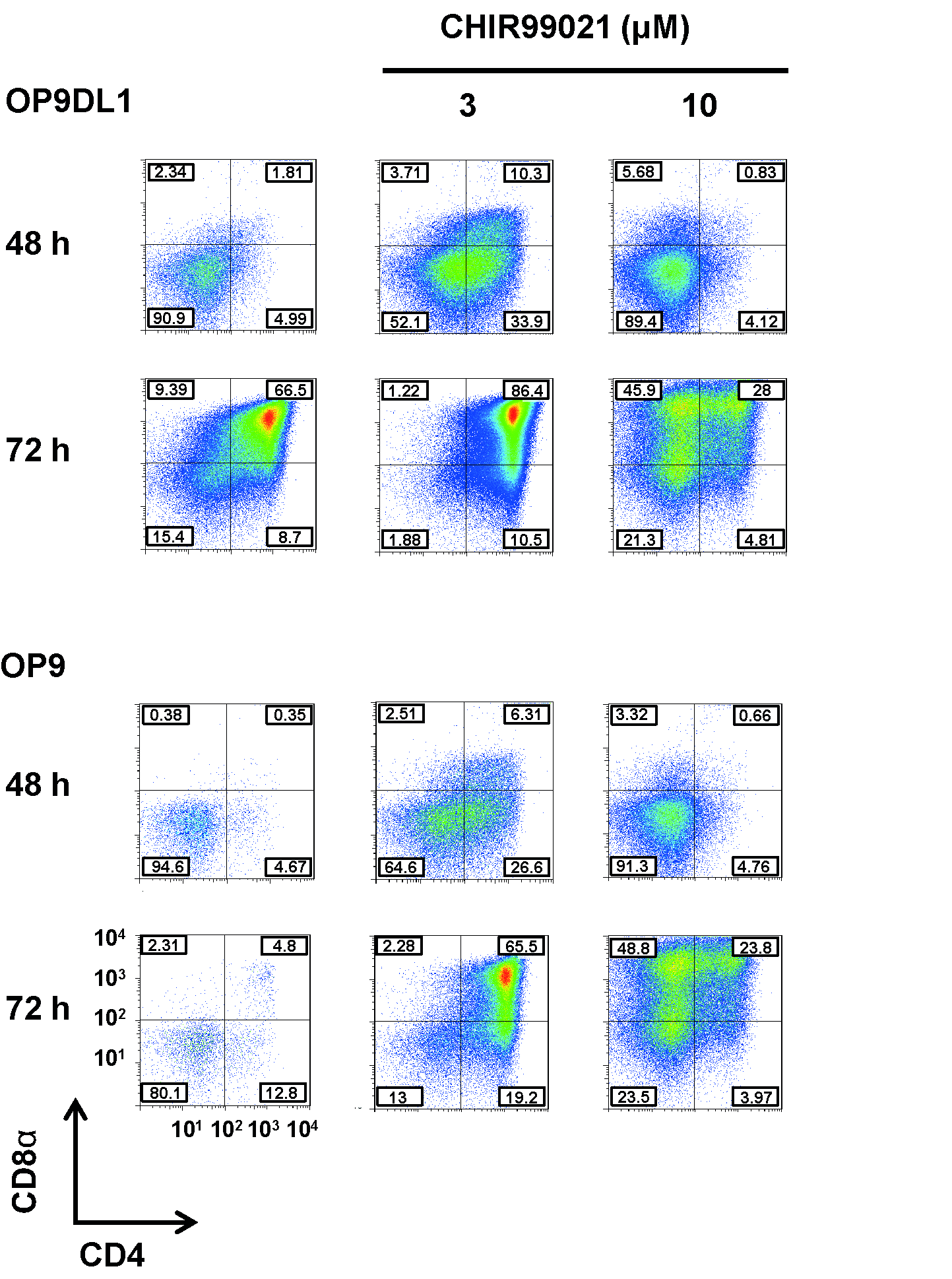

Supplement: Figure S1 — CHIR99021 enhances DN3a development and proliferation in the absence of Notch1 signalling. DN3a cells were cultured on OP9-DL1 or OP9 cells in the presence or absence of CHIR99021 (at 3 or 10 µM). The percentages of CD4+CD8+ (DP), CD4+CD8− (CD4 SP), CD4−CD8+ (CD8 SP) and CD4−CD8− (DN) cells present following 48 and 72 hours of culture were analyzed. (TIF) [file pone.0058501.s001.tif]

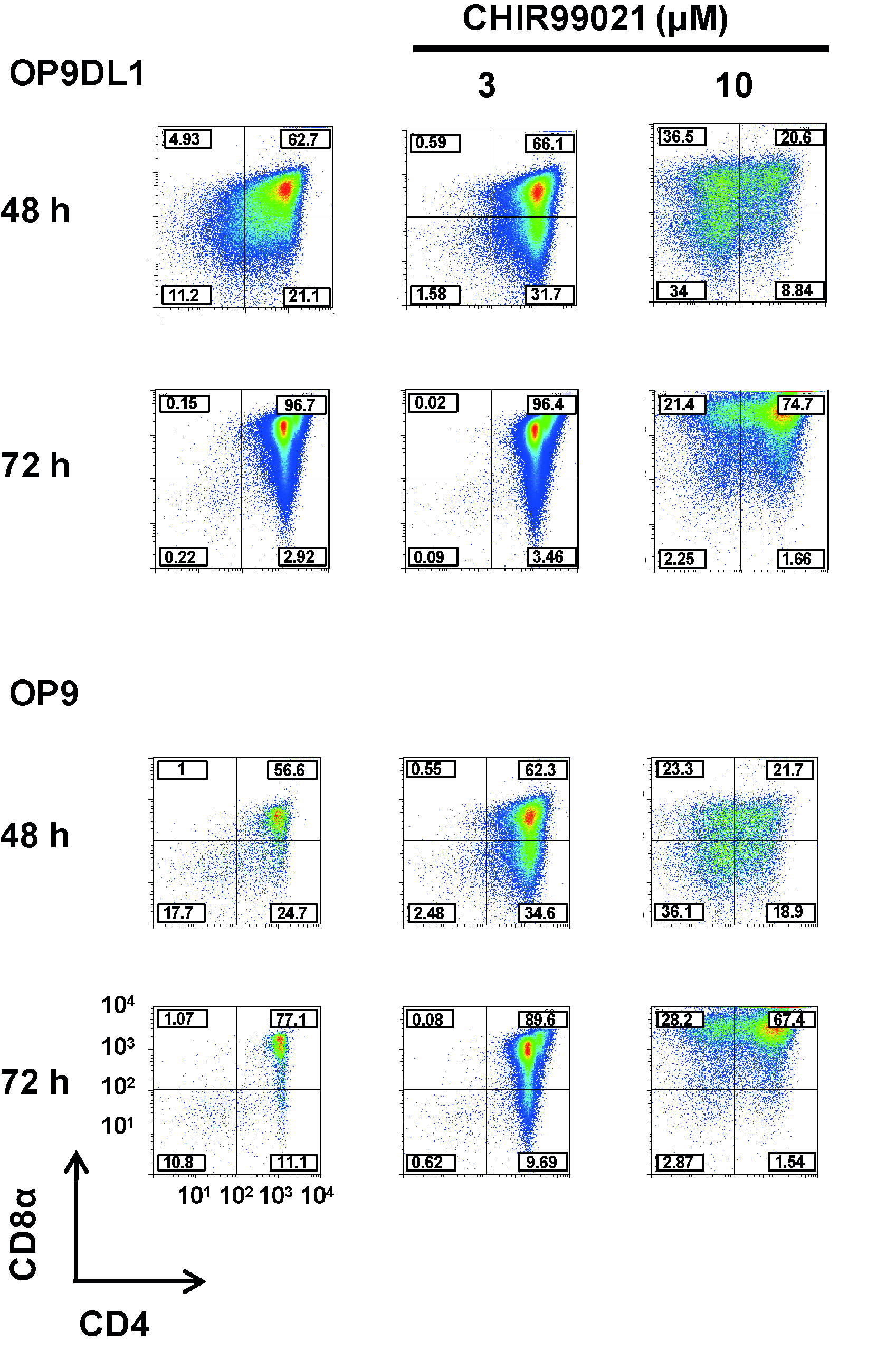

Supplement: Figure S2 — CHIR99021 enhances DN3b development and proliferation in the absence of Notch1 signalling. DN3b thymocytes were cultured on OP9-DL1 or OP9 cells in the presence or absence of CHIR99021 (3 or 10 µM). The percentages of CD4+CD8+ (DP), CD4+CD8− (CD4 SP), CD4−CD8+ (CD8 SP) and CD4−CD8− (DN) cells were analyzed. (TIF) [file pone.0058501.s002.tif]

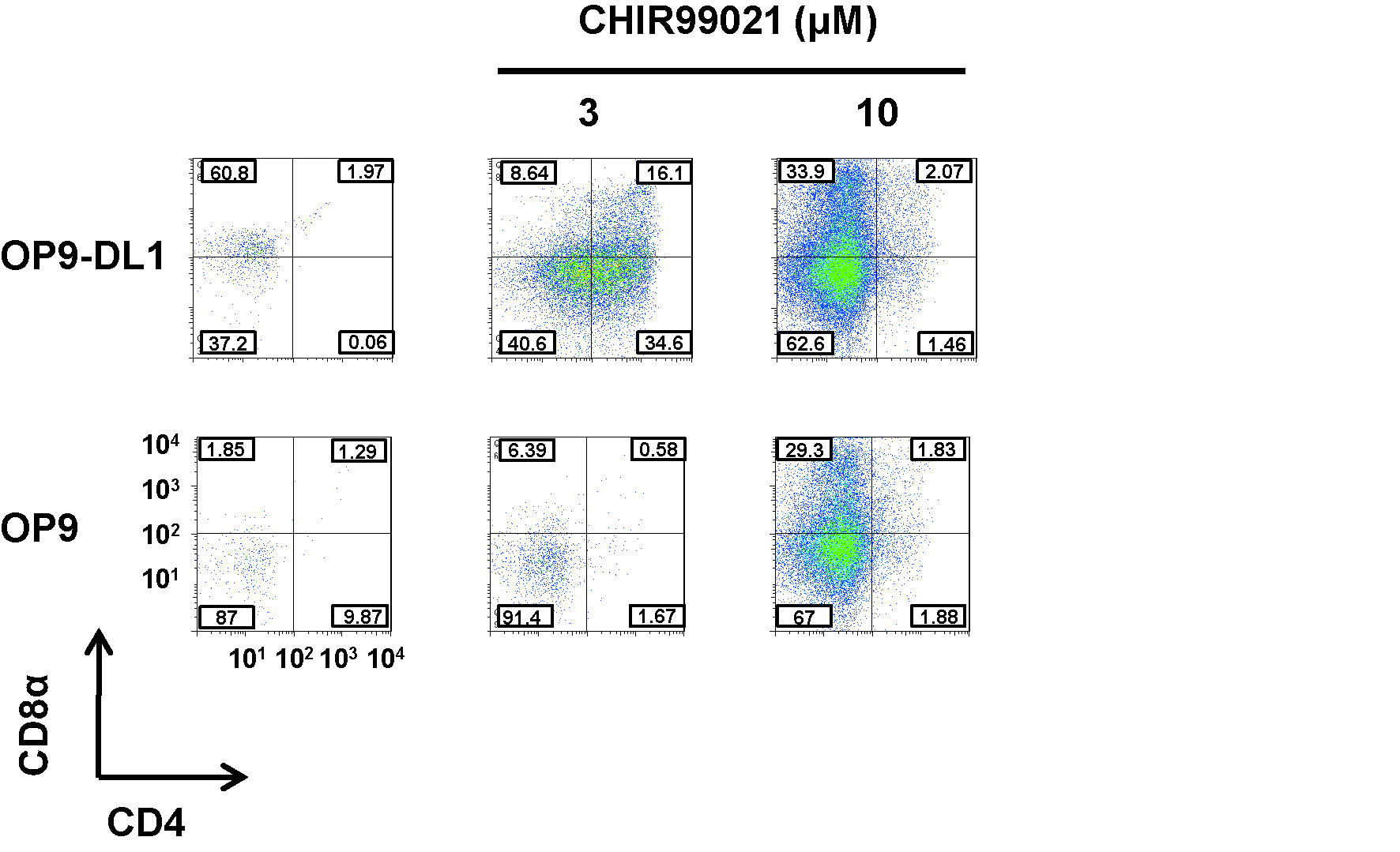

Supplement: Figure S3 — CHIR99021 enhances DN3 development in the absence of preTCR signalling. Rag-2−/− DN3 cells were cultured on OP9-DL1 or OP9 cells in the absence or presence of CHIR99021 (3 or 10 µM). After 72 hours cells were harvested and analyzed for the percentage of CD4+CD8+ (DP), CD4+CD8− (CD4 SP), CD4−CD8+ (CD8 SP) and CD4−CD8− (DN) cells. (TIF) [file pone.0058501.s003.tif]

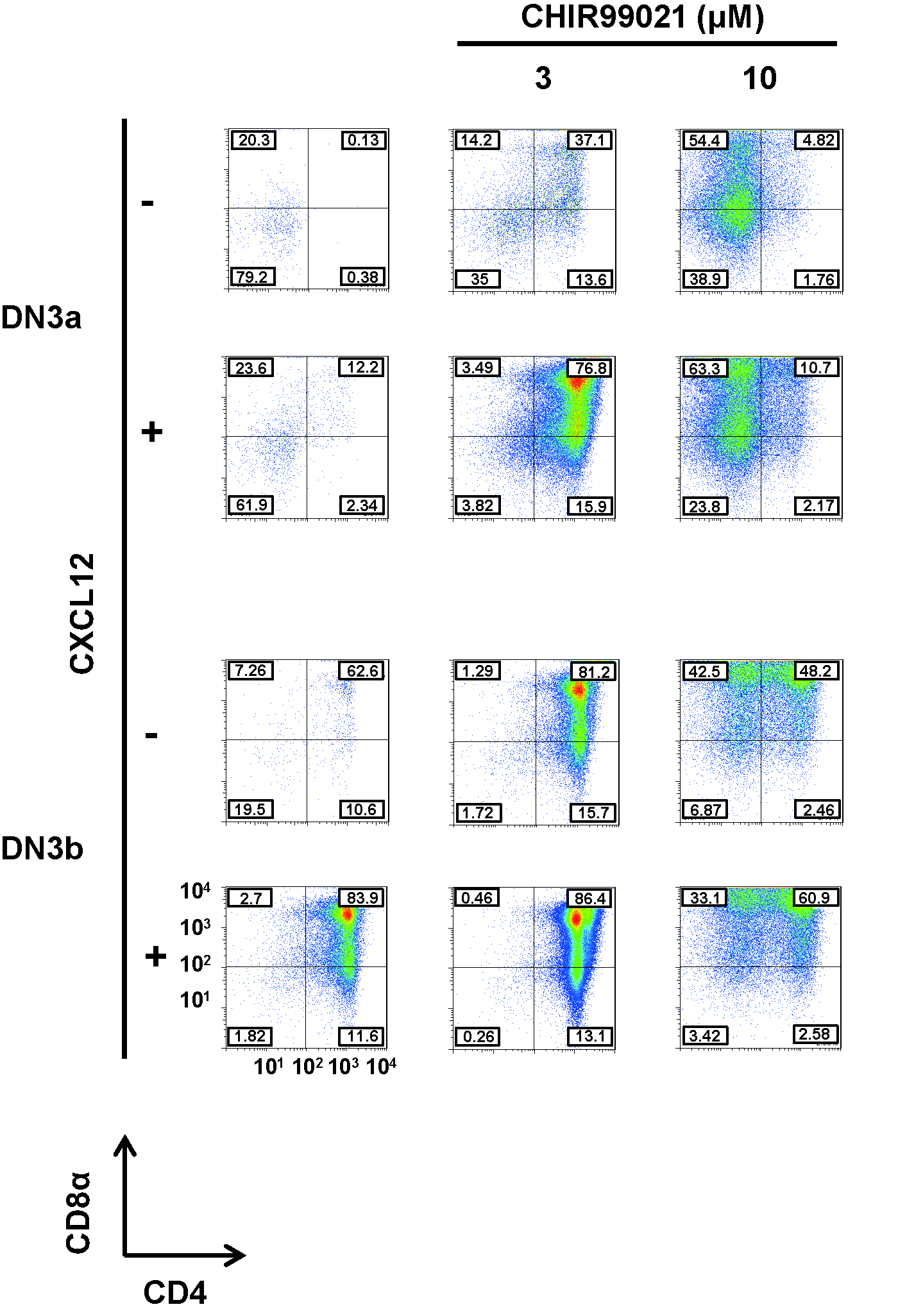

Supplement: Figure S4 — CHIR99021 enhances DN3 development in the absence of CXCL12. DN3 were cultured on plate-bound recombinant DL4 in the presence or absence of CXCL12 (10 nM) and CHIR99021 (1, 3 or 10 µM). After 72 hours cells were harvested and analyzed for the percentages of CD4+CD8+ (DP), CD4+CD8− (CD4 SP), CD4−CD8+ (CD8 SP) and CD4−CD8− (DN) cells. (TIF) [file pone.0058501.s004.tif]

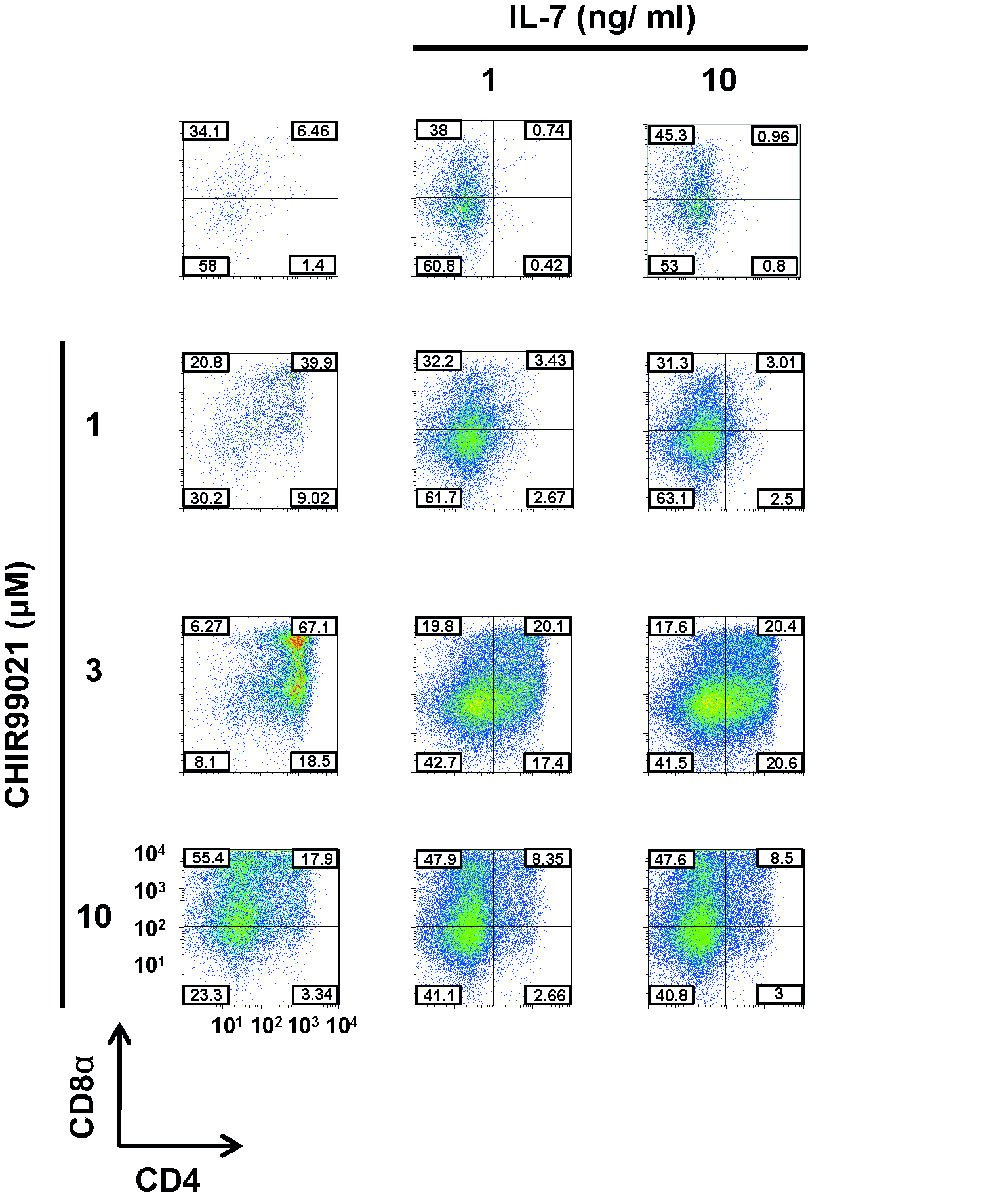

Supplement: Figure S5 — Effects of CHIR99021 and IL-7 on DN3 cell development. DN3a cells were cultured on plate-bound recombinant DL4 in the presence of CXCL12 (10 nM) and the presence or absence of the indicated concentrations of CHIR99021 (1, 3 or 10 µM) and recombinant murine IL-7 (1 or 10 ng/ml). The percentage of CD4+CD8+ (DP), CD4+CD8− (CD4 SP), CD4−CD8+ (CD8 SP) and CD4−CD8− (DN) cells was analysed after 72 hours. (TIF) [file pone.0058501.s005.tif]

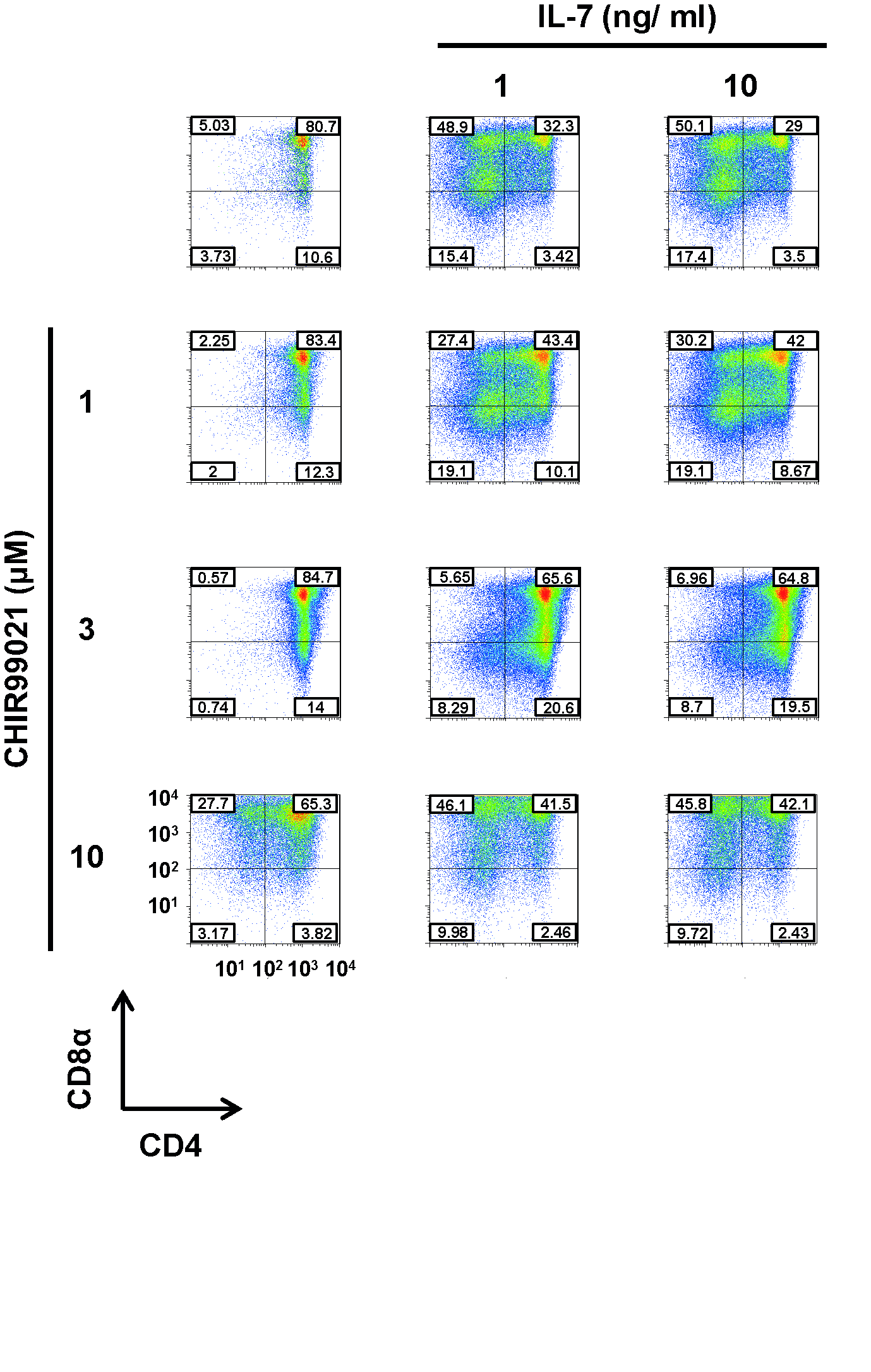

Supplement: Figure S6 — Effects of CHIR99021 and IL-7 on DN3 cell development. DN3b cells were cultured on plate-bound recombinant DL4 in the presence of CXCL12 (10 nM) and the presence or absence of the indicated concentrations of CHIR99021 (1, 3 or 10 µM) and recombinant murine IL-7 (1 or 10 ng/ml). The percentage of CD4+CD8+ (DP), CD4+CD8− (CD4 SP), CD4−CD8+ (CD8 SP) and CD4−CD8− (DN) cells was analysed after 72 hours. (TIF) [file pone.0058501.s006.tif]
