# Supplementary figures and images for: Correction: Pharmacological Inhibition of Glycogen Synthase Kinase 3 Regulates T Cell Development In Vitro
Source: PLoS One. 2013 May 17;8(5):10.1371/annotation/851be907-9f62-420c-92b8-c31681c3bcbe. doi: 10.1371/annotation/851be907-9f62-420c-92b8-c31681c3bcbe (PMC3656667; doi:10.1371/annotation/851be907-9f62-420c-92b8-c31681c3bcbe)

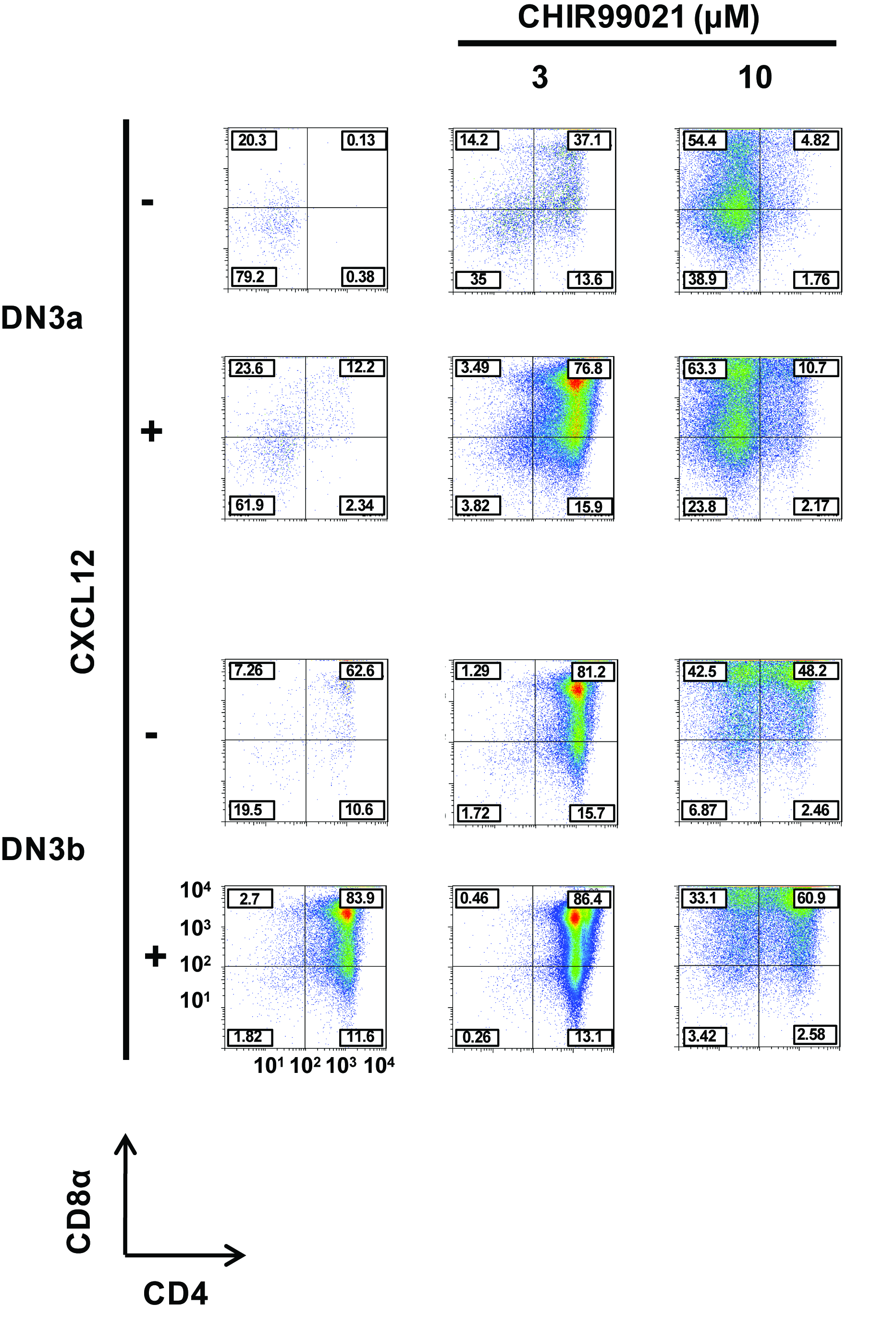

Supplement: Supplementary file 1 [file pone.851be907-9f62-420c-92b8-c31681c3bcbe.s001.tif]

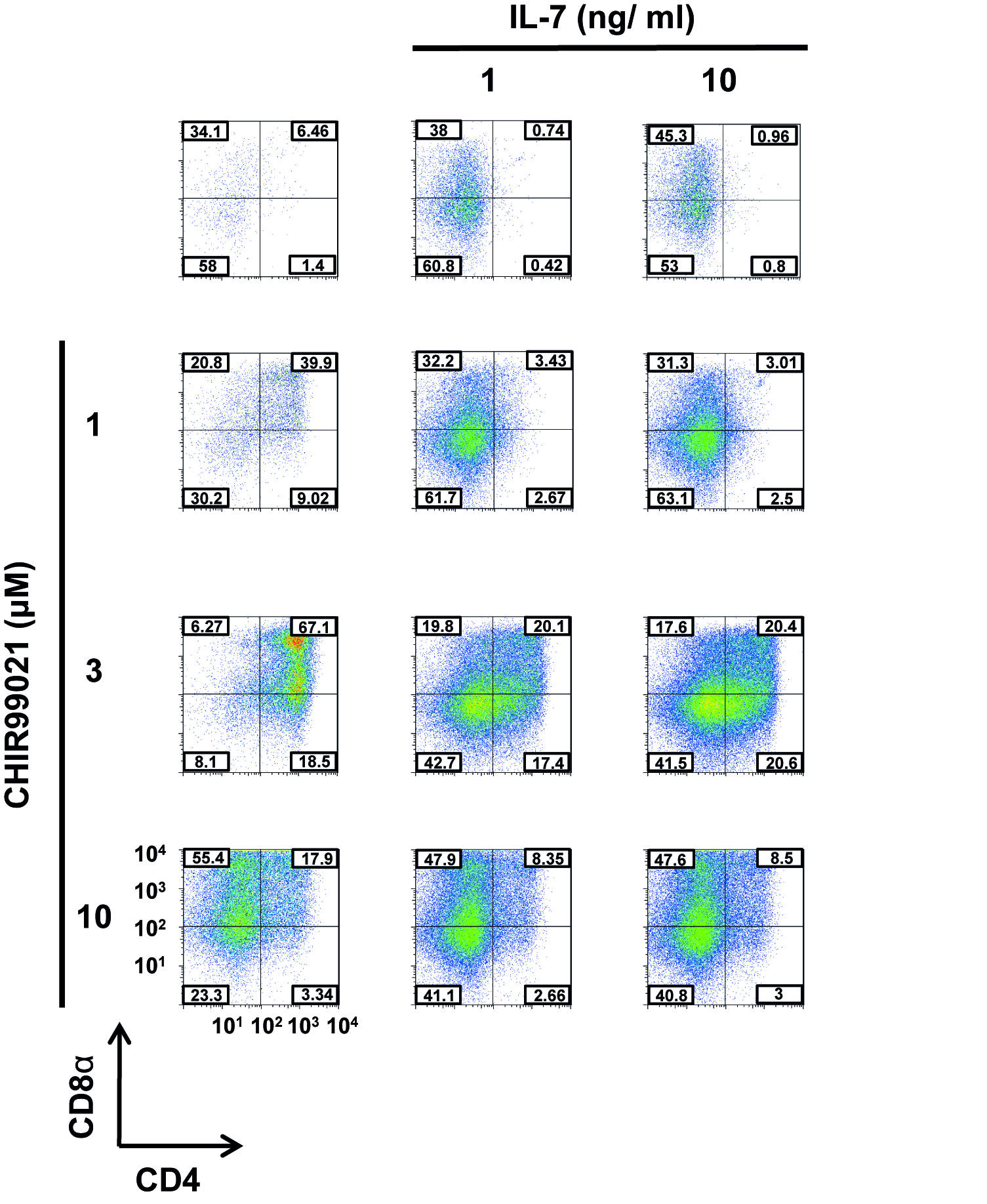

Supplement: Supplementary file 2 [file pone.851be907-9f62-420c-92b8-c31681c3bcbe.s002.tif]

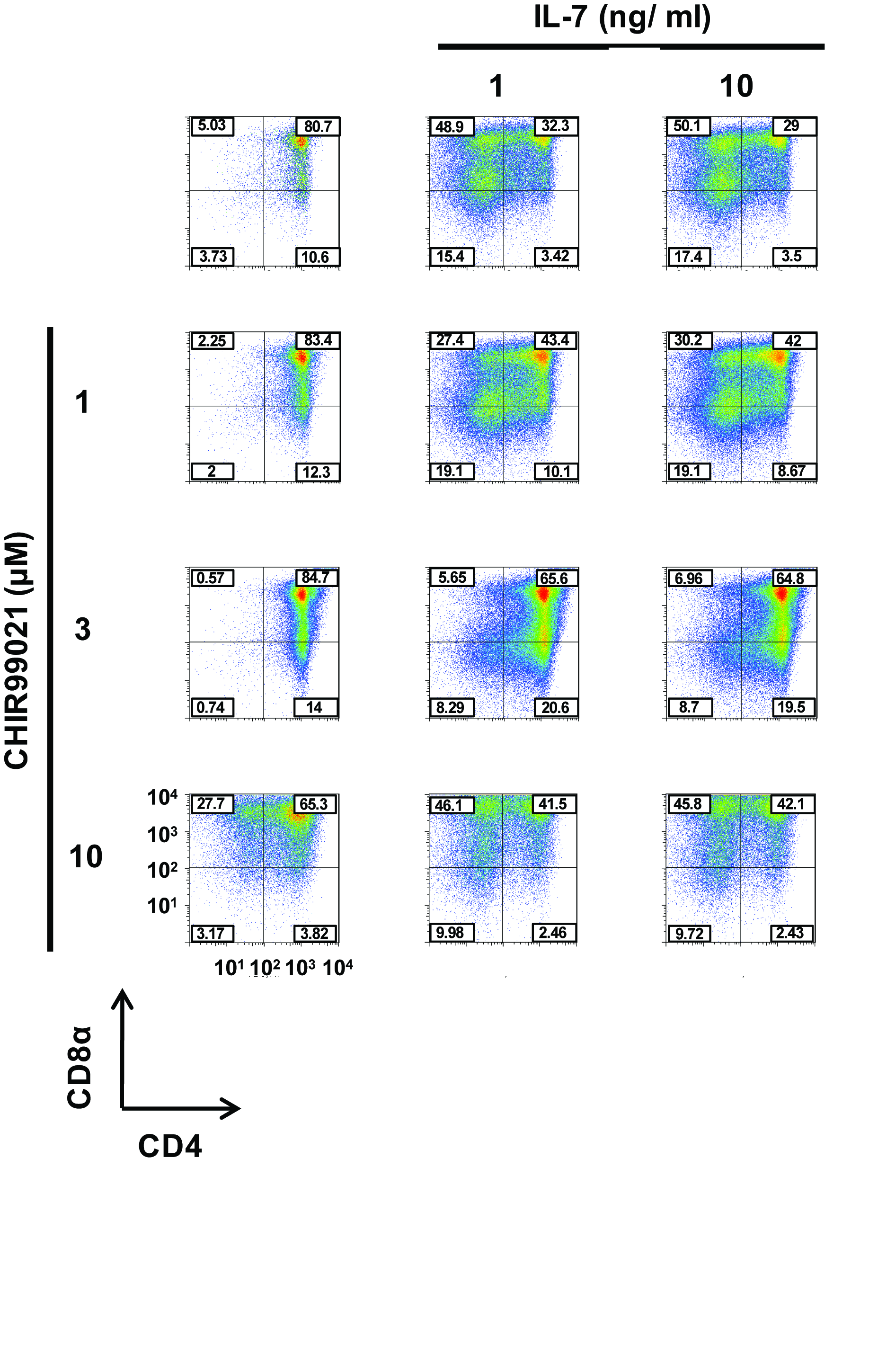

Supplement: Supplementary file 3 [file pone.851be907-9f62-420c-92b8-c31681c3bcbe.s003.tif]
